# Targeting BRCA and DNA Damage Repair Genes in GI Cancers: Pathophysiology and Clinical Perspectives

**DOI:** 10.3389/fonc.2021.662055

**Published:** 2021-10-11

**Authors:** Kai Zimmer, Florian Kocher, Alberto Puccini, Andreas Seeber

**Affiliations:** ^1^ Department of Hematology and Oncology, Comprehensive Cancer Center Innsbruck, Medical University of Innsbruck, Innsbruck, Austria; ^2^ Medical Oncology Unit 1, Ospedale Policlinico San Martino Istituto di ricovero e cura a carattere scientifico (IRCCS), University of Genoa, Genoa, Italy

**Keywords:** BRCA, DNA damage repair, PARP inhibitors (PARPi), precision oncology, molecular profiling, gastrointenstinal cancer

## Abstract

Mutated germline alleles in the DNA damage repair (DDR) genes “breast cancer gene 1” (*BRCA1*) and *BRCA2* have originally been identified as major susceptibility genes in breast and ovarian cancers. With the establishment and approval of more cost-effective gene sequencing methods, germline and somatic *BRCA* mutations have been detected in several cancers. Since the approval of poly (ADP)-ribose polymerase inhibitors (PARPi) for *BRCA-*mutated cancers, *BRCA* mutations gained rising therapeutic implications. The impact and significance of *BRCA* mutations have been evaluated extensively in the last decades. Moreover, other genes involved in the DDR pathway, such as *ATM*, *ATR*, or *CHK1*, have emerged as potential new treatment targets, as inhibitors of these proteins are currently under clinical investigation. This review gives a concise overview on the emerging clinical implications of mutations in the DDR genes in gastrointestinal cancers with a focus on *BRCA* mutations.

## Introduction

The breast and ovarian cancer susceptibility genes “breast cancer gene” (*BRCA*)*1* and *BRCA2* have been thoroughly investigated in the last decades. It is estimated, that one out of 400 to 800 individuals (0.125%–0.25%) in the USA may harbor a germline loss-of-function mutation in *BRCA1* or *BRCA2* (gBRCA-mut) (1, 2). This is associated with an approximately 60% lifetime risk for breast cancer and a 15% to 40% lifetime risk for ovarian cancer development (3–5). Of note, the rate of g*BRCA*-mut is higher in the Ashkenazi Jewish population, in which 2.5% harbor a pathogenic mutation (6). In this specific population, two founder mutations in *BRCA1* and one founder mutation in *BRCA2* have been identified (7).

For *BRCA*-mutated breast and ovarian cancer, treatment with Poly-(ADP) ribose polymerase inhibitors (PARPi) has been established as a standard of care (8, 9). Besides breast and ovarian cancer, PARPi have been recently evaluated for the treatment of *BRCA*-mut pancreatic cancer implicating a possible further use in other *BRCA*-mut gastrointestinal (GI) cancers. The frequency of *BRCA1* and *BRCA2* mutations in GI cancers varies between tumor entities (see **Table 1**). However, as *BRCA1* and *BRCA2* play pivotal roles in the cellular response to DNA damage, targeting DNA damage repair (DDR) mechanisms gained focus in drug development. In addition, as oncologists, it is paramount to be aware of the crucial implications related to the broadening population of patients who are going to be tested for and counseled about germline BRCA mutations, since the recognition of hereditary syndromes is important in order to set up dedicated follow-up to reduce the incidence of second tumors among survivors and to reduce mortality among their relatives (22). As treatment options in advanced GI cancers are limited and are associated with a poor overall survival (OS), the quest towards new therapeutics is ongoing, and drugs targeting *BRCA* and other gene members within the DDR complex are heavily investigated. In this article, we aimed to give a concise overview on the pathophysiology of the DDR machinery, current and upcoming treatment options for GI cancers with *BRCA1/2* mutations, and on other DDR genes that are currently under clinical investigation.

**Table 1:** Mutation frequencies of BRCA1, BRCA2, and of other frequently mutated DDR genes.

Gene	Esophageal (%)	Gastric (%)	Pancreatic (%)	HCC (%)	CRC (%)	BTC (%)
BRCA1	0.48^†^		1.3–1.4^†‡^	s: 0.29^†^–0.6^‡^	0^†^	1.06^†^
				g: 1–7^§^		
BRCA2	s: 2.91^†^		s: 3.1–3.3^†‡^	s: 2.3^†^–3^‡^	0^†^	2.2^†^
	g: 3–12^~^	g: 5.7^**^	g: 6–17^$^
PALB2	s: 0.81^†^		0.6^#^	1.17^†^	0^†^	0.69^†^
ATM	3.23^†^		3.6^†^–18^††^	4.08^†^	0.87^†^	4.57^†^
ATR	0.32^†^		0^†^	0.29^†^	0^†^	0.73^†^
CHK2	0.97^†^		0.6^†^	2.33^†^	4.35^†^	1.3^†^
WRN	0.16^†^		0.12^†^	0.29^†^	0^†^	0.29^†^–1.2^§§^
overall HRR Mut	20.8^†^		15.4^†^	28.9^†^	20.3^†^–22.8^‡‡^	15.0^†^

s, somatic; g, germline; no annotation, somatic mutation; BTC, biliary tract cancer; HCC, hepatocellular cancer; CRC, colorectal cancer; HRR, homologous recombination repair. ^†^Heeke et al. (10), ^§^Ricci et al. (11), ^§§^Zimmer et al. (12), ^‡^Spizzo et al. (13), ^$^Klein et al. (14), ^~^Ko et al (15), Akbari et al. (16), Hu et al. (17), ^**^Figer et al. (18), ^‡‡^Lin et al. (19), ^††^Russel et al. (20), ^#^Seeber et al. (21).

## BRCA Genes Are Key Players in the DNA Damage Repair Complex

DDR mechanisms maintain genomic integrity and stability by restoring DNA damage arising from intracellular and extracellular stressors. Those stressors can lead to base alterations or single- (SSBs) or double-strand breaks (DSBs) (23). If left unrepaired, strand breakages can result in the breakdown of chromosomes and, subsequently, in the loss of genes. Mainly, DNA DSBs cannot only arise from stalled or broken DNA replication forks but can also be caused by ionizing radiation, reactive oxygen species (ROS), and physical or mechanical stress (23).

**Figure 1:**
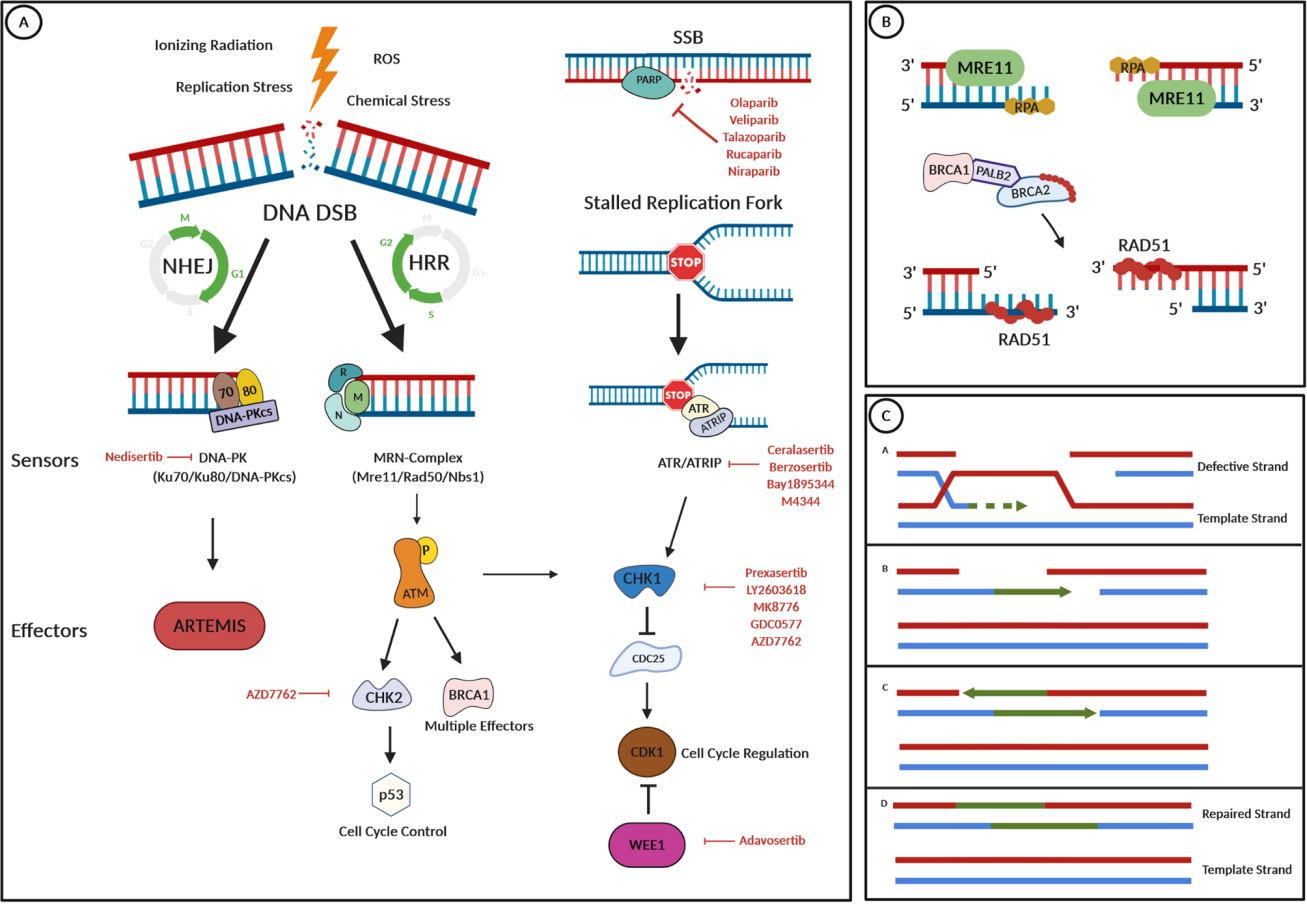
DNA damage repair pathways and drug targets. **(A)** Several internal and external stressors such as ionizing radiation, replication stress, ROS, or chemical stress can lead to DNA double-strand breaks (DNA DSBs). Depending on cell cycle progression and the availability of a template strand, either the nonhomologous end joining (NHEJ) or the homologous recombination repair (HRR) pathway are the mechanism of choice for DNA repair. In the NHEJ pathway, DNA damage is recognized by the “DNA-dependent protein kinase” (DNA-PK), a nuclear serine/threonine protein kinase complex, composed of a large catalytic subunit named DNA-PKcs and a heterodimer of Ku proteins (Ku70/80). Then, the nuclease Artemis is activated and cleaves 5′- and 3′-DNA overhangs. In HRR, which only occurs in the S and the G2 phase of the cell cycle, a protein complex termed MRN (which is composed of MRE11, Rad50, and Nbs1) recognizes DNA DSBs. After its activation by phosphorylation, the ATM kinase works as a master effector protein for HRR by activating several downstream effectors such as the CHK1 or the CHK2 kinase or the BRCA1 protein, having effects on cell cycle progression *via* p53 control or CDK1 inhibition. Stalled replication forks lead to an arrest of DNA replication and such replication stress could lead to DNA DSBs. To prevent this and to stop cell cycle progression, the ATR protein kinase is recruited and binds to its partner protein ATRIP. Cell cycle regulation and repair mechanisms are then controlled *via* CHK1 activation. Repair of DNA single-strand breaks (SSBs) is initiated by PARP enzymes. **(B)** In HRR, the MRE protein acts as a nuclease and resects the broken DNA ends, which results in the overhanging of single-stranded 3′-ends. Protection from further resection or modification of ssDNA is reached by the binding of the RPA protein. RAD51 is essential for HRR, as it facilitates DNA unwinding, stretching, and invasion into the template strand. To prevent its early polymerization on DNA, RAD51 proteins are kept inactive by binding to BRCA2, which is recruited to the broken DNA in a complex with BRCA1 and the linker protein PALB2 after detection of DNA damage. The RAD51-DNA filament is then capable of invading the template strand. **(C)** The RAD51-DNA filament invades the template strand and searches for extended homologous regions, which are then stabilized by base pairing. RAD51 disassembles and leaves a heteroduplex of the defective DNA strand and the template strand. The invading strand is extended by a DNA polymerase, and after elongation, the newly synthesized strand segment is displaced and finally ligated with its original strand endings. This figure was created using the BioRender.com online tool.

Two mechanisms have been discovered which counteract serious DNA damage. Predominantly, cells utilize a mechanism termed “nonhomologous end joining” (NHEJ), in which the broken DNA strands are brought into proximity and the ends are joined by DNA ligation (24). This mechanism is error prone, as processing of the DNA ends by NHEJ leading to the loss of nucleotides and causing rearrangements, which might lead to an altered DNA sequence at the site of breakage. However, if a sister chromatid is available as a template for the repair machinery during or shortly after DNA replication in the S- and G2-phase of the cell cycle (25), NHEJ is avoided and a mechanism termed “homologous recombination repair” (HRR) is preferably used. *BRCA1* and *BRCA2* are important players in the molecular machinery of HRR, that accurately repair DSBs and prevents the delay or arrest of the cell cycle, apoptosis, or the passing on of damaged DNA (26). In brief, HRR repairs DSBs *via* exchange of DNA strands between a pair of homologous duplex DNA sequences, whereas one strand acts as a template to restore the lost or damaged information on the other strand (**Figure 1**).

In the HRR pathway, *BRCA1* acts upstream of *BRCA2* and has multiple distinct functions, which are mediated by its different genetic domains (27), emphasizing the pivotal role of *BRCA1* as a caretaker of the genome, as it directly and indirectly interacts with DNA damage sensors, DDR effectors, tumor suppressors, and cell cycle regulators (28, 29). “partner and localizer of BRCA2” (PALB2) binds directly to both, *BRCA1* and *BRCA2*, and thereby provides a physical link between the two proteins (27, 30, 31).

Lack of a functional *BRCA1* or *BRCA2* allele leads to a deficiency in DNA DSB repair by HRR. In such a constellation, the cell utilizes more error-prone mechanisms such as NHEJ or single-strand annealing, which in turn, leads to increased genomic instability (32). In *BRCA1*-deficient cells, chromosomal aberrations, such as triradial or quadriradial chromosomes, or rearrangements, like translocations, have frequently been observed (33). In *BRCA2*-deficient cells, aneuploidy has been observed due to deregulation of centrosomes and the mitotic spindles (34). An important step in carcinogenesis of *BRCA-*mutated cells seems to be the loss of heterozygosity (LOH), as this can be observed in most gBRCA-mut cancers (35). However, there are data suggesting that loss of the wild-type allele may not be a prerequisite of *BRCA*-associated tumorigenesis (27). Besides LOH, promotor methylation of the *BRCA* genes seems to be an important mechanism for loss of function (LOF) and was more frequently observed in sporadic than in g*BRCA*-mut cancers (36). A *BRCA1* methylation signature has also been associated with a better response to cytotoxic treatment in a small cohort of breast cancer patients (37).

## Platinum-Based Regimens Are Considered Efficient in DDR Aberrant Cancers

Cytotoxic chemotherapeutic agents, such as platinum agents, lead to interstrand crosslinks, resulting in distortion of the DNA double helix, which inhibits the transcription, as RNA polymerases stall at the platinum crosslinks. If left unresolved, the cell evokes a programmed cell death pathway (38). However, in the situation of a hypofunctional DDR, e.g., through a LOF or a hypermethylation of the *BRCA* genes, these lesions are left unrepaired to a greater extend and the viability of cells is reduced dramatically (39). This observation has already been transferred to clinical settings and has been implemented in the treatment of gynecological malignancies. As such, in *BRCA*-mut breast cancer, the use of platinum-based therapeutic regimens, showed promising results in triple negative breast cancer (40–44), and the first randomized phase III trial (TNT trial) led to a longer progression-free survival (PFS) in patients with BRCA mutations compared with *BRCA*-wt patients. However, no effect on OS has been observed (45). Some studies have also reported improved PFS, OS, and response rates (RR) in ovarian cancer patients with *BRCA* mutations undergoing platinum-containing regimens (46–54).

## PARP Inhibitors Generate Synthetic Lethality in HRR Defect Cancers

The development of PARPi added further treatment options for *BRCA*-mut cancers by making use of the concept of synthetic lethality. Synthetic lethality between two genes occurs where individual loss of either gene is compatible with life, but simultaneous loss of both genes results in cell death (55). Endogenous processes (i.e., oxidation) lead to thousands of mutated bases every day, which are mainly repaired by a mechanism called base excision repair (BER). BER generates single-nucleotide gaps (i.e., SSBs), which has to be filled by a DNA polymerase and then be sealed by a DNA ligase (56). The PARP1 protein recruits those DNA repair enzymes to the SSB sites, by binding to these sites of breakage and ADP-ribosylates itself (this has been termed “PARylation”). The resulting poly-ADP tails serves as docking sites for repair enzymes needed to fix the SSBs (57). When PARP1 is blocked, SSBs persist, which generates DSBs when a replication fork passes through (58). In *BRCA-*deficient cells, those DBSs cannot be repaired during replication, leading to cell death by accumulation of DSBs and the resulting genomic catastrophe (55).

However, this classical model (“SSB replication run-off model”) of our understanding of the mechanism of action of PARPi is still incomplete and has been challenged by the “PARP1 trapping model” and the “replication restart model,” which are reviewed elsewhere (55, 59).

Clinical evidence for the efficacy of PARPi in g*BRCA*-mut patients has first been observed in breast and ovarian cancer and was later also observed in metastasized castration-resistant prostate cancer and pancreatic cancer (60). To date, the PARPi olaparib, rucaparib, talazoparib, veliparib, and niraparib gained FDA approval in different settings.

Although its promising activity, more than 40% of *BRCA*-deficient patients fail to respond to PARPi and acquired resistance commonly occurs (60). Four categories of resistance mechanisms have been identified so far (61): cellular availability of the inhibitor, activity and abundance of PAR chains, reactivation of HRR, and replication fork protection. Of these, especially the restoration of HRR genes through genetic reversion mutations in *BRCA1* or *via* synthetic viable loss of *53BP1* has been clinically proven (61–63). Also, resistance to platinum-based chemotherapies strongly predicts PARPi resistance (64).

## BRCA Mutations in Pancreatic Cancer

With an estimated 5-year OS below 5% in the metastasized setting, new therapeutic approaches are urgently needed for patients with pancreatic cancer (65).

A family history of pancreatic cancer is reported in about 5%–10% of newly diagnosed cases (66–68) with germline mutations in *BRCA2* contributing to approximately 6%–17% of these cases (14, 66, 69) (**Table 1**). Also, truncating mutations in *PALB2* were identified to occur in 1%–3% of familial pancreatic cancer cases (70–72). In a large cohort comprising 2,818 sporadic pancreatic cancers, our group identified gene mutations in *BRCA1*, *BRCA2*, or *PALB2* in 1.3%, 3.1%, and 0.6%, respectively. Interestingly, none of the patients with a *BRCA1* or *BRCA2* mutations had a concomitant mutation in *PALB2* and vice versa. *BRCA*-mut and *PALB2*-mut statuses were also associated with a distinct genomic profile, potentially predictive for response to checkpoint-inhibitor therapy (21). Other estimates of *BRCA* gene mutation prevalence in sporadic PDAC ranged from 5.5% to 21.6% (73). Before the discovery of PARPi, several case reports and small clinical trials suggested a benefit for DNA-crosslinking chemotherapy, e.g., with platinum, in *BRCA*-mut pancreatic cancer (74–77). In 2014, Golan et al. retrospectively analyzed clinical data of 71 patients with *BRCA1/2*-mut pancreatic cancer and observed a significantly longer OS for individuals with an advanced disease when treated with a platinum therapy compared with nonplatinum treatment (22 vs. 9 months) (66).

In the last decade, several clinical trials investigated PARPi in *BRCA*-mut pancreatic cancer. In a phase II trial including 23 patients with metastatic pancreatic cancer, the PARPi olaparib reached a RR of 21.7%, a stable disease (SD) ≥8 weeks in 35%, and a median PFS and OS of 4.6 and 9.8 months, respectively (78) (**Table 2**). In the first randomized, placebo-controlled phase III trial evaluating the efficacy of olaparib as a maintenance treatment in patients with g*BRCA1/2*-mut and metastatic pancreatic cancer (POLO trial), a significant prolongation of PFS was observed. For the 92 patients treated in the interventional arm, median PFS reached 7.4 months compared with 3.8 months in the placebo arm (HR, 0.53). However, an interim overall survival analysis failed to show a survival benefit (86). Interestingly, olaparib maintenance therapy was associated with improved quality of life in the POLO trial (90). As such, olaparib has been approved by the FDA as a first-line maintenance treatment in gBRCA-mut metastatic pancreatic cancer. Promising upcoming clinical trials are currently evaluating the efficacy of olaparib in patients with somatic mutations in *BRCA* and other HRR genes (NCT02677038) and a combinational approach with the PD-1 immune checkpoint-inhibitor pembrolizumab (NCT04548752).

**Table 2:** Published clinical trials using PARP inhibitors in pancreatic cancer.

Clinical Trial identifier	Phase	PARP inhibitor	Other intervention	RR	OS/PFS (months)	BRCA-mut status assessed	Addtional information	Reference, year
NCT00515866	I	Olaparib	+Gemcitabine	N.A.	N.A.	n.r.		Bendell et al. (79)
NCT01078662	II	Olaparib	–	ORR 21.7%	SD >8 weeks 35%	gBRCA1/2-mut	Also ovarian, breast, prostate	Kaufmann et al. (78)
NCT00576654	I	Veliparib	+Irinotecan	PR 19%	N.A.	n.r.	Several other solid tumors, also CRC	LoRusso et al. (80)
NCT01296763	I	Olaparib	+Irinotecan +Cisplatin +Mitomycin C	ORR 23%	N.A.	n.r.	1 patient with gBRCA2 mut had a durable response	Yarchoan et al. (81)
NCT01286987	I	Talazoparib	–	PR 15%	N.A.	gBRCA1/2 mut in expansion cohort	Several advanced solid tumors; 1 with PR had BRCA2-mut, 1 haad PALB2-mut	De Bono et al. (82)
NCT02042378	II (RUCAPANC)	Rucaparib	–	ORR 15.8%	N.A.	gBRCA1/2 mut		Shroff et al. (83)
NCT01233505	I	Veliparib	+Capecitabine + Oxaliplatin	1 pt with pancreatic Ca had SD	N.A.	Known BRCA-mut or high probability	Also mCRC, ovarian	Turk et al. (84)
N.A.	II	Veliparib	–	No PR SD 25%	mPFS 1.7 mOS 3.1	Known gBRCA1/2 mut	Also PALB 2 mutations	Lowery et al. (85)
NCT02184195	rIII*	Olaparib	–	ORR 23 vs. 12% (n.s) 2 CR in olaparib	PFS 7.4 vs 3.8 mOS 18.9 vs 18.1	gBRCA1/2 mut	Olaparib as maintenance therapy	Golan et al. (86)
NCT01908478	I	Veliparib	+Gemcitabine +RT		mOS 15 months	n.r.	Subgroup analysis for DDR-mut suggests OS benefit	Tuli et al. (87)
NCT01489865	I/II	Veliparib	+FOLFOX +5-FU	ORR overall 26% in DDR-mut 57%		n.r.	DDR-mut data was used for subgroup analysis	Pishvaian et al. (88)
NCT01585805	rIb/II	Veliparib	+Gemcitabin +Cisplatin	RR (A): 74.1% vs. (B) 65.2%	mPFS (A) 10.1 months vs. (B) 9.7 months, mOS (A): 15.5 months vs. (B) 16.4 months	gBRCA1/2 mut	Arm A + veliparib, B only chemotherapy	O’Reilly et al. (89)

ORR, overall response rate; PR, partial response; SD, stable disease; N.A., not available; n.r., not required for inclusion; gBRCA1/2-mut, germline BRCA 1/2 mutations; (m)PFS, (median) progression free survival; mOS, median overall survival. *Randomized.

In the phase II RUCAPANC trial enrolling 19 *BRCA1/2*-mut pancreatic cancer patients (with three of those harboring a somatic *BRCA* mutation), rucaparib led to response in 15.8% (*n* = 3, 2 PR, 1 CR) of patients (83). Therefore, rucaparib for *BRCA*-mut pancreatic cancer is currently evaluated in further ongoing clinical trials. A phase Ib/II trial evaluates a combinational approach of rucaparib with irinotecan, fluorouracil, and leucovorin in different GI tumors (NCT03337087). The LODESTAR trial (NCT04171700) is a phase II multicenter open-label study of rucaparib as treatment for solid tumors, associated with deleterious mutations in several HRR genes, and another trial investigates rucaparib as a maintenance treatment for patients that have not progressed on platinum-based therapy (NCT03140670).

In 16 patients with known germline *BRCA1/2* mutation receiving the PARPi veliparib in a phase II setting, no partial or complete responses (PR and CR, respectively) was observed. However, four patients had a SD ≥4 months (85).

In 58 patients within a single-arm, open-label phase I/II study, investigating a combination of veliparib with 5-fluorouracil and oxaliplatin, the ORR was 26% (PR: *n* = 11, CR: *n* = 4), PFS was 4.0 months and OS was 7.8 months. One patient showed an exceptional response, remaining in CR for nearly four years. Interestingly, in the subgroup of patients with prior platinum treatment, the ORR was only 7% compared with 32% in those who were platinum naive. This finding suggests that mechanisms of acquired resistance to platinum overlap the mechanisms of resistance to PARPi (88).

In an open-label, randomized, multicenter, two-arm phase II trial, 50 patients with a germline *BRCA1/2* or *PALB2* mutation were randomized to cisplatin and gemcitabine with or without the addition of veliparib. The addition of veliparib resulted in a higher disease control rate (100% vs. 78%), but no prolongation in PFS or OS was observed. The authors concluded that cisplatin and gemcitabine may be a new standard approach in g*BRCA/PALB2*-mut pancreatic cancer (89, 91).

Other promising clinical trials in *BRCA*-mut pancreatic cancer include a phase II trial investigating the combination of the PARPi iraparib and the PD-1 Inhibitor dostarolimab (NCT04493060), a phase I trial investigating the platinum salt BTP-114 in several *BRCA*-mut or other DDR genes mutated solid tumors (NCT02950064) or a phase II trial evaluating the wee1-inhibitor adavosertib (the MATCH Screening trial, NCT02465060) (**Supplementary Tables S1** and **S2**).

Besides the prominent clinical role of BRCA mutations, loss of ATM is an even more common event in pancreatic cancer. This is implicated in the early stages and the progression of PDACs (20) and can be found in about 4% to 18% of cases. To target this alteration, ATR inhibitors are considered effective and are under investigation in currently recruiting clinical trials (**Supplementary Table S3**). A retrospective clinical characterization of 22 ATM-mutated PDACs showed a prognostic value of these alterations compared with control patients with superior 5-year OS rates of 38.3% vs. 6.6% suggesting a possible role as a biomarker (92).

## BRCA Mutations in Other GI Cancers

### Esophageal Cancer

Considering the poor prognosis of esophageal cancer patients, there is an urgent need towards novel therapeutic agents. However, trials evaluating novel treatment have been disappointing so far. In an approach to define molecular subgroups of esophageal cancer, Secrier et al. performed whole-genome sequencing in 129 esophageal cancer samples and established three subtypes with genetic implications for potential stratified and targeted therapeutic approaches. Approximately one in three tumors was classified as “C>A/T dominant” due to a C>A/T mutational pattern. About one-fifth was classified as “DDR impaired”, presenting with defects in HRR and chromosome segregation pathways. Fifty percent of the samples was classified “mutagenic,” as they presented with a dominant T>G mutational pattern and the highest mutational burden and the highest neoantigen load (93). However, in the DDR-impaired subgroup, *BRCA1/2* mutations were rare, and the authors speculated, that a pathway-level disruption of HRR contributes to a *BRCA*-like mutational signature rather than mutations of *BRCA* genes. Large epidemiological studies investigating the incidence of *BRCA* mutations in esophageal cancer and estimating the risk of esophageal cancer in g*BRCA*-mut carriers are limited. However, studies from regions with very high incidence rates in esophageal cancer, found *BRCA2 *mutations in about 3% to 12% in these selected populations (15–17). The frequency of somatic mutations in *BRCA1/2* has been estimated for gastroesophageal cancers with 0.48% for *BRCA1* and 2.91% for *BRCA2* (10) (**Table 1**).

To the best of our knowledge, no results of large clinical trials investigating the role of HRR targeting agents, such as PARPi, in esophageal cancer are available so far. However, patients with esophageal cancer and with deleterious mutations in HRR genes are eligible for the LODESTAR trial (NCT04171700) in which treatment with rucaparib will be evaluated. Ongoing clinical trials will also investigate niraparib (NCT03840967), a combination of olaparib and anti-VEGFR antibody ramucirumab (NCT03008278), olaparib alone (SOLAR trial, NCT03829345), a combination of rucaparib and ramucirumab with or without the anti-PD1 antibody nivolumab (NCT03995017), or a combination of olaparib with paclitaxel and the anti-PD1 inhibitor pembrolizumab (NCT04592211). Of note, screening for HRR prior to inclusion is not a prerequisite for all of these trials (**Supplementary Table S2**).

A study performed in 144 patients with advanced or metastatic esophageal cancer receiving cisplatin- or docetaxel-based treatment, found, that a low *BRCA1* mRNA expression correlated with an increase in RR and median OS in patients treated with cisplatin, but was associated with decreased RR and OS in patients treated with docetaxel (94). This suggests a role of *BRCA1* mRNA expression levels as a predictive and prognostic marker in esophageal cancer.

### Gastric Cancer

Considering the low rate of g*BRCA*-mut carriers with gastric cancer and very limited data on somatic *BRCA* mutations in gastric cancer (**Table 1**), only small clinical trials have been conducted. Clinical data of BRCA-mut gastric cancer patients is therefore also limited, but it is suggested, that this trait might be predictive for treatment with DNA damaging agents (95).

In a subgroup analysis of the MEDIOLA basket trial evaluating the combination of olaparib and the anti-PDL1 antibody durvalumab, an ORR of 10% in gastric cancer patients was reported (96). Of note, in this trial, patients with metastatic gastric cancer were not selected by g*BRCA*-mut status, and no subgroup analysis has been presented.

In 2013, Chen et al. analyzed BRCA1 protein expression by immunohistochemistry (IHC) of 637 gastric cancer samples to evaluate relationships between BRCA1 expression, already established prognostic factors, platinum-based adjuvant chemotherapy, and survival. Positive BRCA1 staining was observed in 34% of patients. Interestingly, BRCA1 positivity was associated with a significant prolongation of survival and BRCA1-negative patients seemed to benefit from platinum-based adjuvant chemotherapy (97). In 318 patients with stage II/III sporadic gastric cancer cases, approximately half of the patients were identified as BRCA1 negative by IHC. BRCA1 negativity seemed to be associated with a reduced disease-free survival but predicted response to adjuvant chemotherapy (98). In a large cohort of 367 patients with sporadic gastric cancer *BRCA1* and *BRCA2* mRNA levels were investigated by IHC, ISH and RT-qPCR. In this study no association with clinical-pathological biomarkers and survival with BRCA status was observed (99).

### Biliary Tract Cancer

Biliary tract cancer (BTC) comprises gallbladder cancer (GBC), intrahepatic (IHCC), and extrahepatic cholangiocellular carcinoma (EHC).

In a molecular profiling approach, 28.9% of BTC tumors harbored pathogenic mutations in several HR-DDR genes (10). Another study found germline or somatic mutations in DDR genes in 63.5% of patients with BTC. Moreover, a significantly prolonged PFS and OS were observed in patients harboring DDR alterations and who received first-line platinum containing chemotherapy (100).

Recently, our group reported results from a NGS study of 1,292 patients with BTC and found *BRCA2* mutations in 3% and *BRCA1* mutations in 0.6%. *BRCA2* mutations were significantly more frequent than *BRCA1* mutations in GBC and IHCC (4.0% vs. 0.3% and 3.7% vs 0.4%). No difference was observed in EHC (2.6% *BRCA2* vs. 2.1% *BRCA1*). Also, a significant association of BRCA-mut carriers with MSI-H/dMMR and higher TMB was observed, suggesting a potential rationale for the combination of PARPi and immune checkpoint inhibitors (13).

Clinical trials in *BRCA*-mut BTC are limited. A small retrospective case series with 18 cases of *BRCA* associated EHCs, of which 13 received platinum-based therapy and four received a PARPi, the authors reported a median OS of 40.3 months for patients with stage I/II BTC, and an OS of 25 months for stage III/IV BTC (101).

To date, no clinical trial on targeting mutations in *BRCA* or other DDR genes in BTC has been presented, but several promising trials are ongoing. For example, a phase II trial evaluates olaparib in BTC with mutations in 16 DDR genes (NCT04042831), whereas another trial compares the combination of olaparib and the ATR inhibitor AZD6738 with the combination of AZD6738 and durvalumab (NCT04298021). The PARPi rucaparib will be evaluated in a phase I/II trial in combination with irinotecan, fluorouracil and leucovorin (NCT03337087), and in another trial in combination with nivolumab (NCT03639935).

### Hepatocellular Carcinoma

Literature involving *BRCA* mutations in hepatocellular carcinoma (HCC) is limited. The BCLC reported a fourfold increase of RR in HCC patients with BRCA mutations compared with BRCA wt patients (68, 102).

In a cohort of 214 patients with HCC, DDR gene alterations were observed in 22.8%, of which 85% harbored a somatic mutation (19). Another study analyzing data from a large industrial molecular profiling company was unable to identify any somatic *BRCA1/2* or *PALB2* mutation in HCC patients, but the overall frequency of mutations in 25 HRR associated genes was estimated with 20%, with *ARID1A* contributing to nearly half of these cases (10).

Despite promising preclinical evidence (103), no larger clinical trial targeting *BRCA*-mut in HCC is currently under way.

### Colorectal Cancer

The risk for colorectal cancer (CRC) in BRCA carriers seems elevated in women below the age of 50 and also for anal carcinoma (104), making screening measures an important tool in this cohort. In a small case series, BRCA-mutated CRCs were also found to be more often of mucinous histology (105), which is a feature also associated with other defects in DNA repair genes such as the mismatch repair genes suggesting a distinct tumor biology and underlining the need for further investigation.

However, to date, no larger clinical trials targeting somatic *BRCA*-mut in CRC patients have been published. In several small clinical trials investigating PARPi in CRC, *BRCA* status has not been assessed as an eligibility criterion and only one upcoming trial (LODESTAR, NCT04171700) will select CRC patients according to *BRCA*-mut status.

In a small study in CRC patients, monotherapy with PARPi resulted in an ORR of 0% (106). However, used in combination with chemotherapy or radiotherapy, ORR rose up to 57% in a study combining veliparib with FOLFIRI with or without bevacizumab (107). No stratification according to *BRCA*-mut status was performed.

## Beyond BRCA and PARP Inhibitors in GI Cancers

In the currently largest approach to identify DDR gene alterations in GI cancers, 17,486 patients with GI cancer were screened and at least one alteration in a subset of 10 DDR genes was identified in 17.1%, with gastric cancer being the most frequently DDR mutated tumor (27.1% had at least one DDR gene mutated), providing a rationale for clinical trials (108) in this specific subset.

Besides PARPi, emerging DDR associated targets in GI cancers include *ATM*, *ATR*, *CHK1/2*, *WRN*, or *WEE1*. *ATM* plays a pivotal role as a master regulator of DNA damage recognition and repair (**Figure 1**) and it is among the most commonly aberrant genes in sporadic cancers, especially in hematologic malignancies (109). Heterozygosity for a germline *ATM*-mut can be observed with a frequency of approximately 1% in the population and increases cancer risk by two- to threefold (110, 111). Frequency of somatic mutations in GI cancers ranges from 0.87% in HCC to 4.5% in CRC (111), making it a considerable druggable target. To date, the ATM inhibitor AZD0156 is the first-in-class agent to be investigated in a phase I clinical trial (AToM trial, NCT02588105) in patients with advanced malignancies including gastric cancer as a monotherapy or in combination with olaparib or with irinotecan/FOLFIRI. Preclinical studies suggested a potentiated effect of olaparib when used in combination with AZD0156 (112). A randomized, double-blind phase II trial investigating the combination of olaparib and paclitaxel stratified patients by ATM protein levels and found a longer OS for the addition of olaparib regardless of the ATM expression levels (113) (**Supplementary Table S3**). In this trial, signals for a longer OS in the population with low ATM expression led to further analysis in the phase III GOLD trial. Unfortunately, the GOLD trial did not meet its primary endpoint of a significant improvement in OS with the addition of olaparib, neither in the overall population (8.8 vs. 6.9 months) nor in the ATM-negative population (12.0 vs. 10.0 months) (114). Biomarker subgroup analysis (i.e., for BRCA) of the GOLD-trial cohort, was unable to identify any significant association with clinical outcomes (115).

Preclinical data support the hypothesis that loss of *ATM* leads to increased sensitivity to ATR inhibition (116). Also, preclinical data suggest a dependency on ATR signaling in PARPi-resistant *BRCA*-mut cells for replication fork stabilization (117–119), providing a rationale to combine ATR inhibitors (ATRi) with PARPi to overcome and prevent secondary resistance mechanisms. Currently, ATRi (AZD6738, VX-970/M6620, M4344, BAY1895344) are investigated in several phase I and phase II trials involving GI cancers (**Supplementary Table S1**). Combinational approaches include crosslinking agents, PARPi or PD-1/PD-L1 inhibitors. One trial investigates the combination of AZD6738 in combination with olaparib in patients with IDH-mutant cholangiocellular carcinoma (NCT03878095). Another phase II trial investigates the combination of VX-970 and irinotecan in gastric or gastroesophageal junction cancers harboring*TP53* mutations. To the best of our knowledge, DDR mutational status (such as *ATM*) is only assessed in two clinical trials investigating ATRi (NCT04095273, NCT04266912) prior to inclusion.

The combination of AZD6738 (ceralasertib) and paclitaxelin heavy pretreated cancer patients yielded clinical efficacy with an ORR of 25% according to a phase I trial (120). Another phase I trial (NCT02264678), including pancreatic and gastric cancers patients the combination of ceralasertib with olaparib or durvalumab, showed promising preliminary signals of antitumor activity (121). Encouraging safety was found for M6620 (VX-970) as monotherapy or in combination with carboplatin in patients with advanced solid tumors. Of note, one patient with metastatic colorectal cancer, harboring a loss in *ATM* and an *ARID1A* mutation, achieved a CR and had an ongoing PFS of 29 months (122). M6620 in combination with gemcitabine showed promising results in preliminary data coming from an phase I trial (123).

The CHK1 kinase has been recognized as one of the key components of cell cycle checkpoint response after DNA damage and is essential for HRR (124). Interestingly, in a study using data from a large industrial molecular profiling provider, no somatic *CHK1* mutation was observed in GI cancers, but a prevalence of somatic *CHK2* mutations between 0.6% in pancreatic cancer and 4.35% in HCC was observed (10). To date, several CHK1/2 inhibitors are under investigation for clinical use. In a phase I trial including patients with advanced solid tumors monotherapy with the CHK 1/2 inhibitor prexasertib (LY2606368) resulted in a PR rate of 4.4% activity was observed (PR 4.4% comparably, the CHK1 inhibitor GDC-0575 in combination with gemcitabine showed only modest clinical efficacy in a phase 1 trial including 102 patients with several solid tumors with only four patients achieving PR (3.9%) (125).

The WEE1 nuclear kinase is a key regulator of cell-cycle progression (126) and is phosphorylated by CHK1 upon DNA damage. Subsequently, WEE1 leads to the inactivation of the “cyclin-dependent kinase 1” (CDK1)-cyclin B complex, resulting in cell cycle arrest in the G2 phase (**Figure 1**) (126). Overexpression of WEE1 has been reported in several cancers (126). In pancreatic cancer, preclinical models suggest a combinational approach of a WEE1 inhibitor with radio- and chemotherapy, and a phase I/II trial treated 34 patients with locally advanced PDAC with the WEE1 inhibitor adavosertib (AZD1775) in combination with gemcitabine and radiation therapy. The study found a median PFS of 9.4 months, a median OS of 21.4 months and was well tolerated (127).

In the MATCH screening trial (NCT02465060), patients with a *BRCA1* or *BRCA2*-mut will receive adavosertib. Adavosertib also sensitizes *TP53*-deficient tumor cells to DNA damage and has already shown antitumor activity in a phase I trial involving 202 patients with refractory solid tumors (128). In gastric (129) and colon cancer (130), WEE1 seems to be overexpressed and is therefore a suggested target in these entities.

Preclinical data support combinational approaches of PARPi with ATRi or WEE1i to overcome resistance mechanisms and sensitize tumors to radiotherapy (RT) (119, 131). A phase I clinical trial investigates such an approach in esophageal cancer by adding adavosertib to RT (NCT04460937). The phase I STAR trial (NCT04197713) investigates a combination of adavosertib with olaparib in patients with germline or somatic mutations in several HRR genes. However, the combination of WEE1i and PARPi is associated with high rates of toxicity, but toxicity might be reduced by a sequential dosing approach (132). Also, the combination of an ATRi and a WEE1i produced synthetic lethality in a preclinical model and supports further clinical investigation (133).

For the NHEJ machinery, DNA-PK is an important mediator and is also investigated as a druggable target. An inhibitor of this kinase, nedisertib, is combined with RT in localized pancreatic cancer (NCT04172532), and in combination with the anti-PD-L1 inhibitor avelumab and RT, among others, in BTC (NCT04068194).

Emerging in the field of DDR targets is the Werner syndrome helicase (WRN) that has been associated with a distinct molecular landscape in CRC and was associated with higher TMB, higher prevalence of MSI-H/dMMR and PD-L1 expression, supporting further trials investigating the use of immune checkpoint inhibitors. Also, WRN inhibitors are under preclinical investigation, but those results have not been transferred to clinical trials, so far (12).

Rationales for other combinational treatments have been reviewed extensively elsewhere (119, 134, 135).

## Discussion and Future Perspectives

Here, we presented a review on the clinical perspectives of targeting DDR pathways in GI cancers with a focus on *BRCA*.

While the role of *gBRCA* mutations for the lifetime risk of several cancers has been evaluated extensively over the last two decades, the understanding and epidemiology of somatic *BRCA* mutations and other DDR genes in carcinogenesis, and further, specific treatment of such cancers, is still in an early phase. The risk of developing a specific GI tumor in individuals with a *gBRCA* mutation varies between entities and between certain *BRCA* mutations (i.e., B*RCA1* and *BRCA2*). For g*BRCA2*-mut carriers, the risk of developing pancreatic, esophageal, gastric, and BTC is higher than in g*BRCA1*-mut carriers. For HCC, risks seem comparable, and for CRC, evidence is unclear if g*BRCA1*-mut carriers have a higher lifetime risk for CRC compared with g*BRCA2*-mut carriers (68, 102). The highest estimated lifetime risk for g*BRCA2*-mut carriers was reported in BTC (fivefold) (68),. This tissue tropism raises fundamental questions on the biology of the respective tissues both in health and carcinogenesis, suggesting different vulnerabilities to intra- and extracellular stressors or environmental factors.

Intensive follow-up and screening for breast cancer has been established in *gBRCA*-mut carriers, but the value of screening for other cancers, such as GI tumors apart of CRC, is controversial. In pancreatic cancer, for patients with an *ATM*, *BRCA2* or *PALB2* mutation, guidelines recommend, that yearly screening should start at age 50 or 10 years younger than the youngest relative with pancreatic cancer, preferably by MRI/MRCP and/or EUS (136). Such imaging procedures would also allow to screen for BTCs. However, consensus was not reached for screening in *BRCA1*-mut carriers. In CRC, recommendations are not that clear, but clinicians should offer screening at age 40 in *BRCA*-mut carriers with a first-degree relative with CRC or advanced adenoma (137). In gastric cancer, guidelines recommend a prophylactic total gastrectomy between age 20 and 30 for individuals carrying pathogenic variants in the E-Cadherin (*CHD1*) gene, which is associated with a very high lifetime risk for gastric cancer (138). However, no consensus recommendations on screening for gastric cancer in *gBRCA*-mut carriers have been established, which is also true for gastroesophageal cancer (139). Nevertheless, obtaining a detailed family history should be part of every first counseling in newly diagnosed cancers as it could have important implications for the patient and his or her relatives.

While clinical trials targeting the HRR system provide limited evidence for advantage of the use of PARPi in pancreatic cancer (86), such evidence is lacking for other GI cancers. Also, a *BRCA*-mut status has not been approved as a predictive biomarker, such as MSI for pembrolizumab, which would, i.e., allow a site-agnostic use of PARPi. Some authors suggest that *BRCA1*/2 mutations are not suitable as site-agnostic biomarkers for PARPi therapy as dependency on *BRCA*-mut for tumorigenesis differs between tumor lineages, *BRCA1* and *BRCA2* are merely a small part of the complex phenotype of “BRCAness,” and resistance mechanisms, which restore *BRCA* function, make *BRCA* mutations not an ideal site-agnostic marker. However, an accurate estimate of HRR deficiency may be a better predictive biomarker for upcoming clinical trials (140, 141).

In the future, an increasing number of clinical trials will not only focus on *BRCA* mutations, but on a group of genes defining BRCAness. The concept of BRCAness describes HRR defects in the absence of g*BRCA1/2* mutations, but includes somatic *BRCA* mutations and mutations in several other genes such as *PALB2*, *ATM*, *ATR*, *CHEK1/2*, *ARID1A*, *RAD51*, *NBS*, etc. (33). Interestingly, a BRCAness genotype seems to predispose cellular sensitivity to PARPi and platinum therapy (33), having critical implication for upcoming clinical trials by providing a rationale to screen for a BRCAness phenotype, rather than screening solely for *BRCA1* or *BRCA2* mutations. However, exact and consensual definitions of BRCAness have still to be determined (11).

Also, other prognostic biomarkers for PARPi are heavily investigated. HRR-deficient (HRD) tumors seem to prioritize more error-prone DNA repair pathways, such as NHEJ, resulting in the accumulation of small-insertion deletions and LOH events (142). The assessment of HRD comprises different approaches, as a uniform definition has not been made until today. This makes it challenging to compare different trials using different approaches and lead the path for clinicians. In general, HRD testing relies on either the direct detection of genomic perturbations associated with genomic instability (such as mutational or methylation testing of BRCA or other HRR pathway genes), on the use of surrogates of genomic instability by detecting a genomic scar or on functional assays. Mainly used as indirect measures are the LOH determined by SNP-Sequencing, telomeric allelic imbalance (TAI), and large-scale state transitions (LST) for which each HRD score has been developed (143–145). Currently, for clinical routine use, only two prospectively validated and commercially available tests for assessment of HRD status are available (146): the myChoice CDx (Myriad Genetics) calculates a score based on all three genomic instability features and also includes BRCA1/2 mutations. In contrast, the FoundationOne CDx (Foundation Medicine) only calculates the percentage of genomic LOH. However, the clinical use of those tests is limited to ovarian cancer patients as a companian diagnostic for treatment with PARP-inhibitors and therefore no consensus recommendation for the use in other settings are available at the moment. Also, the value can be limited, as scars in the DNA (e.g., through DNA damaging chemotherapy) might be misinterpreted as HRR deficiency (62). To the best of our knowledge, no general consensus on the nomenclature of the genomic phenotype of BRCAness or “HRDness” has been established so far. Distinct definitions to achieve coherence in publications and clinical trials are desirable.

There is a certain rationale for combined treatment with PARPi and immune checkpoint inhibitors coming from several preclinical studies (147). It is suggested that DDR defects lead to a higher neoantigen load and TMB (12, 108). Moreover, PARPi lead to a more inflamed tumor microenvironment by activating the cGAS-STING pathway (147–149) and by increasing immune cell infiltration. PARPi were also found to upregulate PD-L1 (147, 150), which strongly suggests the combination with anti-PD-1/PD-L1 inhibitors. A phase 1a/b trial investigating the PARPi pamiparib and the anti-PD1 antibody tislelizumab in patients with advanced solid tumors, found the combination to be well tolerated and the ORR was 20% (151). In pancreatic cancer, combination with niraparib and dostarolimab (NCT04493060) and olaparib and pembrolizumab (NCT04548752) are currently under investigation. For other GI cancers, several PARPi/ICPi combinations (**Supplementary Table S3**) are currently evaluated.

Loss of the DNA damage sensor *ATM* has been associated with a survival benefit in platinum-treated CRC patients (62, 152), and preclinical studies suggest treatment of *ATM*-deficient tumors with a PARPi and ATRi combination (153–156). Such an approach could be applied to gastric cancers, in which *ATM* mutations are quite common, while *BRCA* mutations are rare (135, 157). Of note, the combination of a PARPi with chemotherapy (GOLD trial) did not result in longer OS in an *ATM*-low subpopulation but addition of an ATR-inhibitor-improved efficacy. In the setting of BTC, a phase I trial investigates the combination of olaparib and the ATR-inhibitor AZD6728 with or without the addition of durvalumab (NCT04298021). However, in this trial, DDR mutational status is not assessed before treatment.

Looking in the near future, results of several clinical trials, of which only some are mentioned above, are awaited to clear the view on the value of DDR targeting drugs in GI cancers. To date, limited and early clinical data are not sufficient to make a prognosis on the success of this approach and only an empirical and therefore long-lasting approach to the best combinational treatment will lead the path.

## Conclusion

Frequency of germline and somatic *BRCA*-mut and other HRR genes in GI cancers varies extensively between sites, and besides *BRCA*-mut PDAC, clinical implications of such findings must be determined. Soon, various clinical trials will add further evidence for the use of HRR targeting agents in GI cancers. Also, investigating the BRCAness phenotype rather than *BRCA1* or *BRCA2* alone, is gaining more attention and is supported by evidence of promising combinational treatments. Oncologists must not forget the crucial implications related to finding *gBRCA* mutations both for the patients and their relatives.

## Author Contributions

AS, FK, and KZ contributed to manuscript conception. KZ wrote the first draft of the manuscript. AS, FK and AP wrote sections of the manuscript. All authors contributed to the article and approved the submitted version.

## Conflict of Interest

The authors declare that the research was conducted in the absence of any commercial or financial relationships that could be construed as a potential conflict of interest.

## Publisher’s Note

All claims expressed in this article are solely those of the authors and do not necessarily represent those of their affiliated organizations, or those of the publisher, the editors and the reviewers. Any product that may be evaluated in this article, or claim that may be made by its manufacturer, is not guaranteed or endorsed by the publisher.
