# Supplementary material for: Targeting BRCA and DNA Damage Repair Genes in GI Cancers: Pathophysiology and Clinical Perspectives
Source: Front Oncol. 2021 Oct 11;11:662055. doi: 10.3389/fonc.2021.662055 (PMC8542868; doi:10.3389/fonc.2021.662055)
Supplement: Supplementary file 1 [file Table_1.docx]

***Supplementary Material***

1. **Supplemental Tables**

**Supplemental Table 1: Ongoing Clinical Trials investigating the DDR Pathway in Pancreatic Cancer**

| Clinicaltrials.gov identifier | Phase | PARP-Inhibitor | | Other Intervention | Estimated End | Additional Information |
| --- | --- | --- | --- | --- | --- | --- |
| NCT02950064 | I | | ----- | +BTP-114 | 2020 | Breast, ovarian, pancreatic, prostate cancer  BRCA or other DDR mutation |
| NCT02677038 | II | | Olaparib | ----- | 2022 | Somatic BRCA mutations only + mutations in other HRR genes |
| NCT03337087 | Ib/II | | Rucaparib | +Irinotecan +Fluorouracil +Leucovorin | 2022 | Metastatic Pancreatic, Colorectal, Gastroesophageal, biliary |
| NCT03140670 | II | | Rucaparib | ----- | 2022 | also PALB2 mutation, maintenance, no prior progression on platinum |
| NCT04171700 | II | | Rucaparib | ----- | 2022 | Solid tumors with various HRR mutations (LODESTAR Trial) |
| NCT02465060 | II | | ------ | +Adavosertib | 2022 | The MATCH Screening Trial |
| NCT04182516 | I | | NMS-03305293 | ----- | 2022 | Breast, ovarian, pancreatic cancer |
| NCT04493060 | II | | Niraparib | +Dostarolimab (PD-1 Inh) | 2022 | also PALB2 mutations |
| NCT04150042 | I | | ------- | +Melphalan, +BCNU, +Hydroxycobalamin, +Ascorbic Acid, +Ethanol, +autologous hematopoieitic stem cells | 2022 |  |
| NCT04548752 | II | | Olaparib | +Pembrolizumab | 2025 | a gBRCA1/2-mut is required for inclusion |

gBRCA1/2-mut: germline BRCA 1/2 mutation, DDR: DNA Damage Repair

**Supplemental Table 2:** **Ongoing Clinical Trials investigating the Use of PARP inhibitors Other than Pancreatic Cancer**

| ClinicalTrials Identifier | Entity | Phase | PARP-Inhibitor | Additional Intervention | Estimated End | Addtional Information |
| --- | --- | --- | --- | --- | --- | --- |
| NCT03838406 | Gastric cancer | N.A. | ----- | +FOLFOX or +CAPOX | Completed, Results N.A. | Observational, evaluation of the incidence of BRCA loss and association with outcome and prognosis |
| NCT03061058 | Gastric | III | ----- | +Docetaxel, +Oxaliplatin, +Cisplatin, +Irinotecan, +Pemetrexed, S1 | Completed, Results N.A. | Intraperitoneal vs systemic chemotherapy, stratified by BRCA1 mRNA levels |
| NCT02033551 | Gastric, Colon, others | I | Veliparib | ±Carboplatin and Paclitaxel or +FOLFIRI | Completed, Results N.A. | For veliparib mono: BRCA mutation assessed |
| NCT00576654 | Colon, Pancreatic, | I | Veliparib | +Irinotecan | 2020 |  |
| NCT03008278 | Gastric, gastroesophageal junction | I/II | Olaparib | +Ramucirumab | 2021 | HR-mut not inclusion criteria but outcome strata |
| NCT02921256 | Rectal | rII* | Veliparib | +Pembrolizumab + Combination chemo, ET | 2021 |  |
| NCT03840967 | Met esophageal/Gastroesophageal | II | Niraparib |  | 2022 | LOH high status and/or deleterious alterations in HR pathway genes; either somatic or germline mutations |
| NCT03829345 | Esophagogastric | II | Olaparib |  | 2022 | SOLAR Trial, HRR-mut no inclusion criteria |
| NCT04592211 | Gastric, esophageal | Ib/II | Olaparib | +Pembrolizumab +Paclitaxel | 2022 | Somatic HRR mutations and MSS status |
| NCT04511039 | CRC, Gastroesophageal | I | Talazoparib | +Trifluridine/Tipiracil | 2022 | No HRR-mut status assessed |
| NCT02734004 | Ovarian, Breast, SCLC, Gastric | I/II | Olaparib | +MEDI4736, +Bevacizumab | 2022 | BRCA-mut is not mandatory for gastric cancer patients |
| NCT03337087 | Pancreatic, CRC, Gastroesophageal, BTC | I/II | Rucaparib | +Irinotecan +Fluorouracil +Leucovorin | 2022 |  |
| NCT03639935 | BTC | II | Rucaparib | +Nivolumab | 2022 | No BRCA-mut status assessed |
| NCT04298021 | BTC | II | Olaparib | +AZD6728 (ATR inhibitor) or AZD6738+Durvalumab | 2022 | No DDR-mut assessed |
| NCT04166435 | CRC | II | Olaparib | +Temozolomide | 2022 | MGMT promotor hypermethylation required |
| NCT03851614 | CRC, PDAC, Leiomyosarcoma | II | Olaparib | +Durvalumab +Cediranib +Durvalumab | 2022 | DAPPER trial No DDR-mut assessed |
| NCT04042831 | Biliary | II | Olaparib |  | 2023 | 16 abberant DDR gene mutations can be included |
| NCT03995017 | Gastric and Esophageal Adenocarcinoma | I/II | Rucaparib | +Ramucirumab +Nivolumab | 2024 | 50 % unselected cohort, 50 % with HRR-mut status |
| NCT02264678 | Gastric and other solid | I | Olaparib | +Ceralasertib, +Carboplatin, +Durvalumab | 2024 | Gastric cancers will be stratified by ATM pro- and deficient |
| NCT03983993 | CRC | II | Niraparib | +Panitumumab | 2024 | RAS-wt only |
| NCT02678182 | Oesophago-gastric | II | Rucaparib | +Capecitabine, +MEDI4736, +Trastuzumab, +Ramucirumab | 2025 | Randomized, PLATFORM trial, maintenance treatment; maintenance therapy, no HRR-mut status assessed |
| NCT04456699 | CRC | III | Olaparib | ± Bevacizumab vs Bevacizumab+5-FU | 2027 |  |

*r: randomized, N.A.: not available, CRC: Colorectal Cancer, BTC: Biliary Tract Cancer, PDAC: Pancreatic Ductal Adenocarcinoma, SCLC: Small Cell Lung Cancer

**Supplemental Table 3: Clinical Trials targeting DDR Pathway Proteins**

| Target | Drug | ClinicalTrials.gov Identifier | Phase | Additional intervention | DDR-mut status assessed | RR | OS/PFS (months) | Addidtional Information | Reference/  Year |
| --- | --- | --- | --- | --- | --- | --- | --- | --- | --- |
| ATR | **Ceralasertib**  **(AZD6738)** | NCT02630199 | I | +Paclitaxel | n.r. | ORR 25.5 %  1 CR (1.9 %), 12 PR (23.5 %), 18 SD (35.3 %) | N.A. |  | Lee et al., 2020 (105) |
|  |  | NCT03669601 | I (ATRiUM) | +Gemcitabine | n.r. | N.A. | N.A. |  | ongoing |
|  |  | NCT03682289 | II | +Olaparib | ARID1A BAF250a status  ATM-loss | N.A. | N.A. |  | ongoing |
|  |  | NCT03780608 | II | +Durvalumab | ATM pro-/deficient | N.A. | N.A. |  | ongoing |
|  |  | NCT03878095 | II | +Olaparib | IDH1/IDH2 mutant | N.A. | N.A. |  | ongoing |
|  |  | NCT02264678 | Ib | +Olaparib or  +Durvalumab | ATM status  BRCA mut Ov-Ca | Olaparib: 1 CR (2.5 %)  5 PR (12.8 %)  Durvalumab: 1 CR (4.7 %)  2 PR (9.5 %) | N.A. |  | Krebs et al., 2018 (106) |
|  |  | NCT02576444 | II (OLAPCO) | 1: Olaparib  2: Olaparib+Capivasertib*  3: Olaparib+AZD1775**  4: Olaparib+Ceralasertib | 1: IDH1/2, HDR genes^§^  2: PTEN, PIK3CA,AKT, ARID1a  3: TP53, KRAS  4: HDR genes^§^ | N.A. | N.A. | 1: CCC only | ongoing |
|  | **Berzosertib**  **(VX-970/**  **M6620)** | NCT02723864 | I | +Veliparib+Cisplatin | n.r. | 3 PR (13.6 %)  12 SD (57 %) | N.A. |  | O’Sullivan et al, 2018 (148) |
|  |  | NCT03641313 | II | +Irinotecan | TP53-mut required | N.A. | N.A. | Gastric and gastroesophageal cancer only | ongoing |
|  |  | NCT04266912 | I/II (D-BoB) | +Avelumab | DDR deficiency^§§^ | N.A. | N.A. |  | ongoing |
|  |  | NCT03309150 | I | +Carboplatin+Paclitaxel | n.r. | 1 CR (6 %)  5 SD (29 %) | Pat with CR had PFS of 29 months |  | Yap et al., 2020 (107) |
|  |  | NCT02157792 | I | A:+ Gemcitabine+Cisplatin B:1+Cis+Eto B2:+Irinotecan  C1:+Gemcitabine  C2: +Cisplatin, C3:+Cis+Carbo | n.r. | 4 PR (8 %)  2 SD (0.04 %) | PFS 8.3/29.3^+^ | Data only for C1 group | Plummer et al., 2016 (108) |
|  |  | NCT02595931 | I | +Irinotecan | n.r. | N.A. | N.A. |  | ongoing |
|  | **BAY1895344** | NCT04535401 | I | +Irinotecan +Fluorouracil +Leucovorin | n.r. | N.A. | N.A. | Stomach or intestinal cancer | ongoing |
|  |  | NCT04616534 | I | +Gemcitabine | n.r. | N.A. | N.A. | advanced pancreatic or ovarian | ongoing |
|  |  | NCT04491942 | I | +Cisplatin or  +Cisplatin+Gemcitabine | n.r. | N.A. | N.A. | BTC, esophageal, gastric cancerns | ongoing |
|  |  | NCT04095273 | I | +Pembrolizumab | ATM loss/delATM, other DDR genes | N.A. | N.A. |  | ongoing |
|  |  | NCT03188965 | I | ----- | DDR defects, ATM loss | N.A. | N.A. |  | ongoing |
|  | **M4344** | NCT02278250 | I | Carboplatin | LOF in ARID1A , ATRX, DAXX or ATM | N.A. | N.A. |  | ongoing |
| ATM | **AZD0156** | NCT02588105 | I (AToM) | ±Olaparib vs  ±Irinotecan/FOLFIRI | n.r. | N.A. | N.A. |  | ongoing |
| CHK1 | **Prexasertib**  **(LY2606368)** | NCT02124148 | I | A: +Cisplatin vs  B:+Cetuximab vs  E: Samotolisib*** | n.r. | N.A. | N.A. | B:KRASwt CRC only, E: Breast Cancer only | Completed, Results N.A. |
|  |  | NCT03495323 | I | Lodapolimab**** | n.r. | N.A. | N.A. |  | ongoing |
|  |  | NCT02514603 | I | ----- | n.r. | 8 SD (66 %) | N.A. |  | Iwasa et al., 2018 (149) |
|  |  | NCT01115790 | I | ----- | n.r. | 2 PR (4.4 %)  15 SD (33.3 %) | PFS 1.2-6.7 (SD patients) | In dose expansion squamous cell cancers only | Hong et al. 2016, (150) |
|  | **LY2603618** | NCT01341457 | I | +Gemcitabine | n.r. | N.A. | N.A. |  | N.A.^1^ |
|  | **MK8776** | NCT00779584 | I | ±Gemcitabine | n.r. | 2 PR (6 %)  13 SD (43 %) | PFS >19 months in 1 patients with CCC |  | Daud et al. 2015 (151) |
|  | **GDC0577** | NCT01564251 | I | ±Gemcitabine | n.r. | 4 PR (3.9 %) | 15 % had SD > 4 months | 3/4 with PR had TP53mut | Italiano et al., 2018 (110) |
| CHK1/2 | **AZD7762** | NCT00413686 | I | +Gemcitabine | n.r. | 2 PR (4.7 %)  9 SD (23.6 %) | 5 had SD for 6-12 Weeks, 4 had SD >12 weeks | Both PR were NSCLC | Sausville et al., 2014 (152) |
|  |  | NCT00937664 | I | ±Gemcitabine | n.r. | 5 SD (25 %) | SD ranged from 86 to 185 days | all SD were Lung Cancer | Seto et al. 2013 (153) |
| WEE1 | **Adavosertib (AZD1775)** | NCT04462952 | I | ----- | n.r. | N.A | N.A |  | ongoing |
|  |  | NCT04460937 | I | +RT | n.r. | N.A | N.A | Esophageal and Gastroesophageal cancers only | ongoing |
|  |  | NCT04197713 | I (STAR) | +Olaparib | A:germline or somatic BRCA1/2-mut  B:BRCA1/2, BRIP, FANCA, PALB2, ATM, cyclin E mut | N.A | N.A | Patients with PARPi resistance; sequential dosage of olaparib and adavosertib | ongoing |
|  |  | NCT02037230 | II | +Gemcitabine +RT | n.r. | N.A. | PFS: 21.7  OS: 9.4 | Locally advanced pancreatic cancer | Cuneo et al., 2019 (112) |
|  |  | NCT03284385 | II | ----- | n.r. | N.A | N.A | Only patients with a loss of SETD2 | ongoing |
|  |  | NCT04439227 | II (MATCH) | ----- | BRCA1/2-mut | N.A | N.A | Subprotocol Z1 | ongoing |
|  |  | NCT03253679 | II | ----- | n.r. | N.A | N.A | CCNE1 amplification | suspended^2^ |
|  |  | NCT01827384 | II (MPACT) | +Carboplatin | LOF in DDR genes^§§§^ | N.A | N.A | Experimental Regimen II | ongoing |
| DNA-PK | **Nedisertib/**  **Peposertib**  **(M3814)** | NCT04172532 | I/rII | Phase I: +RT  Phase II: +RT vs RT+Placebo | n.r. | N.A | N.A | Localized pancreatic cancer | ongoing |
|  |  | NCT04068194 | I/II | A: RT+Avelumab  B: +RT+Avelumab | n.r. | N.A | N.A | Mainly hepatobiliary cancers but also other solid tumors | ongoing |

LOF: Loss of Function; § ATM, CHK2, APOBEC, MRE11 complex §§ ARID1A, ATM, ATR, ATRX, BAP1, BARD1, BRCA1/2, BRIP1, CDK12, CHEK2, FANCA, FANCC, FANCD2, FANCE, FANCF, FANCM, MRE11A, MSH2, NBN (NBS1), PALB2, RAD51, RAD51C, RAD51D, SMARCB1, and VHL, or other related genes at the discretion, germline mutations in DDR genes are also eligible of the principal investigator; §§§ ATM, ATR ,ERCC1, MLH1, MSH2, NBN, PARP1, PARP2, RAD51, TP53 ; *Capivasertib (AZD5363): Akt-Inhibitor **AZD1775: WEE1 Inhibitor;*** Samotolisib (LY3023414):Pi3K-Inhibitor;****Lodapolimab (LY3300054):anti-PD-L1 antibody; ^+^non-randomized comparison of cohorts 1-4 (VX-970 < 90mg/m2 with Gem 875 mg/m2) and 5-7 (VX-970 ≥ 90mg/m2 with Gem 500mg/m2), n.r. not required prior inclusion; 1: Study results have been published on clinicaltrials.gov but no peer-reviewed publication was available; 2: Trial has been suspended as a protocol amendment is required for expansion
